# Characterization and Bioactivity of Polysaccharides Obtained from Pine Cones of *Pinus koraiensis* by Graded Ethanol Precipitation 

**DOI:** 10.3390/molecules18089933

**Published:** 2013-08-19

**Authors:** Pan Zou, Xin Yang, Wei-Wei Huang, Hai-Tian Zhao, Jing Wang, Ren-Bo Xu, Xing-Long Hu, Si-Yan Shen, Di Qin

**Affiliations:** 1 School of Food Science and Engineering, Harbin Institute of Technology, Harbin 150090, Helongjiang, China; E-Mails: zoupan0601@163.com (P.Z.); 15146748480@126.com (W.-W.H.); zhaoht9999@163.com (H.-T.Z.); xurenboelephant@163.com (R.-B.X.); long_0302@163.com (X.-L.H); xiaoningmeng923@163.com (S.-Y.S.); qidi1990@163.com (D.Q.); 2 Key Laboratory of Agro-Product Quality and Safety, Institute of Quality Standard & Testing Technology for Agro-Products, Chinese Academy of Agricultural Sciences, Beijing 100081, China; 3 Key Laboratory of Agrifood Safety and Quality, Ministry of Agriculture, No. 12 Zhongguancun South Street, Haidian District, Beijing 100081, China

**Keywords:** pine cones, *Pinus koraiensis*, polysaccharides, graded ethanol precipitation, chemical characterization, bioactivity

## Abstract

*Pinus koraiensis* polysaccharides (PKP) were extracted by hot water from *P. koraiensis* pine cones. Five polysaccharide fractions named PKP-A, PKP-B, PKP-C, PKP-D and PKP-E were successfully separated at final ethanol concentrations of 30%, 50%, 60%, 70% and 80%, respectively. HPLC, FT-IR, GC-MS and automatic amino-acid analysis were applied to investigate their chemical characteristics. Monosaccharide component analysis indicated that the five fractions were all composed of d-ribose, l-rhamnose, l-arabinose, d-xylose, d-mannose, d-glucose and d-galactose, but their molar ratios were quite different. HPLC results revealed that the polysaccharides precipitated by higher concentrations of ethanol solution had lower molecular masses. Moreover, the antioxidant activities of the five fractions were studied on the basis of hydroxyl radical and ABTS radical scavenging tests. The five graded polysaccharide fractions exhibited good inhibitory power, and MTT tests *in vitro* showed the IC_50_ of PKP-A and PKP-E were 1,072.5 and 2,070.0 μg·mL^−1^, respectively. These results demonstrated that the PKP could be a potential source of natural antioxidants or dietary supplements.

## 1. Introduction

*Pinaceae Pinus* is commonly found in Manchuria in China, and possesses considerable development potential. Pines are long-lived trees, and the cones accumulate on the forest floor for many years resisting decay [1]. Only a small amount of pine cones is burnt as firewood, and this makes it a waste of biological resources. The Materia Medica Compendium recorded that pine cones have been used historically as tonifying agents for the treatment of respiratory diseases such as excess production of phlegm and asthma for thousands of years in traditional Chinese medicinal prescriptions [2]. Sakagami reported that the main applications of pine cones for clinical treatment are to prevent diseases like coughing, enteritis, neurasthenic and suppress viral infection and tumor cells [3]. Therefore, the increasing interest in pine cone polysaccharides is driven by the need to expand the applications of plant bioactive components. It was reported that water extract of Japanese pine cones have therapeutic anti-tumor, anti-HIV and antibacterial properties [4]. Patrick also described that the water extract of *P. pinaster* can improve immunity [5]. Many healing properties are attributed to *Pinus koraiensis* polysaccharides, one of the main active ingredients of *P. koraiensis*. The biological activities of the polysaccharides from pine cones were studied in some reports [6,7]. According to their data, the crude polysaccharides from pine cones of *P. koraiensis* exhibited effective scavenging activities on ABTS radical and hydroxyl radical. In addition to serving as stores of energy and structural components [8], polysaccharides also have various biological activities, which have drawn researchers’ attention.

We focused on the studies of chemical compositions from pine cones of *P. koraiensis*. The essential oil [9] and polysaccharides [7] were extracted from pine cones of *P. aramandii*, *P. koraiensis* and *P. sylvestris var. mongolica* have been investigated [9,10,11,12,13]. However, to the best of our knowledge, no purification and structural characterization of polysaccharides from pine cones of *Pinus koraiensis* has been reported by other groups. In the last few years, polysaccharides have been fractionated by stepwise ethanol precipitation, replacing almost all of the purification protocols that include at least one such step [14]. Therefore, a graded ethanol precipitation was first used to fractionate the crude polysaccharides from pine cones of *P. koraiensis* in order to obtain a deeper insight into the diversity of *P. koraiensis*. The polysaccharides obtained were characterized by HPLC, automatic amino-acid analysis and FT-IR in terms of molecular weights and chemical compositions. Moreover, we investigated the antioxidant activities on scavenging hydroxyl radical or ABTS radical, inhibition activity on HepG2 of each graded polysaccharide fraction.

## 2. Results and Discussion

### 2.1. Isolation, Purification and Composition of the Polysaccharides

The crude polysaccharides were isolated from hot water extract of pine cones of *P. koraiensis*, and then protein was removed by trichloroacetic acid. Five distinct fractions named PKP-A, PKP-B, PKP-C, PKP-D and PKP-E were successfully obtained separately by graded ethanol precipitation. The yields of precipitates were 11.53%, 2.08%, 4.71%, 7.34% and 3.33% collected at the ethanol concentrations of 30%, 50%, 60%, 70% and 80%, while no polysaccharides was precipitated at the concentration of 40%. The molecular weights were estimated in reference to standard dextrans. The average molecular weights (Mw) of PKP-A, PKP-B, PKP-C, PKP-D and PKP-E were 6,881.6, 6,267.2, 5,753.5, 5,518.5 and 5,120.1 kDa, respectively. The main chemical compositions were determined and are given in Table 1. For the polysaccharide fractions obtained, significant differences in chemical compositions and molecular weights were observed. The HPLC results showed that a rise in ethanol concentration led to a decrease of the molecular weight of the polysaccharides. Thus, these results indicated that the high concentration of ethanol solutions favored precipitation of the smaller molecular parts of polysaccharides, a result also confirmed by Xue *et al.* [15].

**Table 1:** Yield, molecular weight, content of neutral sugar and protein of PKPs (*n* = 3).

	PKP-A	PKP-B	PKP-C	PKP-D	PKP-E
Yield (%)	11.53	2.08	4.71	7.34	3.33
Molecular Weight (kDa)	6881.6	6267.2	5753.5	5518.5	5120.1
Neutral Sugar (%)	44.33 ± 0.15	42.67 ± 0.15	66.33 ± 0.15	58.33 ± 1.85	67.33 ± 1.23
Uronic acid (%)	87.87 ± 0.52	82.88 ± 0.78	53.27 ± 0.13	50.59 ± 0.78	45.06 ± 1.56
Polyphenol (%)	3.45 ± 0.10	1.20 ± 0.05	0.43 ± 0.02	0.43 ± 0.00	0.82 ± 0.09
Protein (%)	2.10 ± 0.15	1.03 ± 0.03	0.48 ± 0.02	0.50 ± 0.01	0.76 ± 0.06

Table 2 showed the amino acid content of the five purified polysaccharides determined by an automatic amino-acid analyzer, where notable differences were found. Asp was not detected in PKP-C, PKP-D and PKP-E. Compared with other fractions, the content of most amino acids was much higher because of the high protein content. 

**Table 2:** Content of amino acid of five purified PKPs (μg·mg^−1^).

	PKP-A	PKP-B	PKP-C	PKP-D	PKP-E
Asp	1.400	0.353	Not detected	Not detected	Not detected
Thr	1.023	0.433	0.957	1.027	1.347
Ser	1.291	0.503	0.882	1.275	1.527
Glu	1.479	0.432	0.079	0.100	0.119
Gly	0.846	0.282	0.233	0.255	0.363
Ala	0.684	0.231	0.305	0.448	0.547
Cys	1.254	0.362	0.349	0.215	0.234
Val	1.131	1.285	0.656	1.032	1.244
Met	1.416	0.071	0.813	0.076	0.046
Ile	0.439	0.138	0.047	0.084	0.112
Leu	0.622	0.154	0.073	0.068	0.099
Tyr	0.804	0.241	0.215	0.120	0.161
Phe	0.597	0.163	0.082	0.063	0.071
Lys	0.313	0.127	0.137	0.121	0.134
His	0.196	0.050	0.044	0.032	0.048
Arg	0.250	0.066	0.044	0.030	0.068

**Figure 1:**
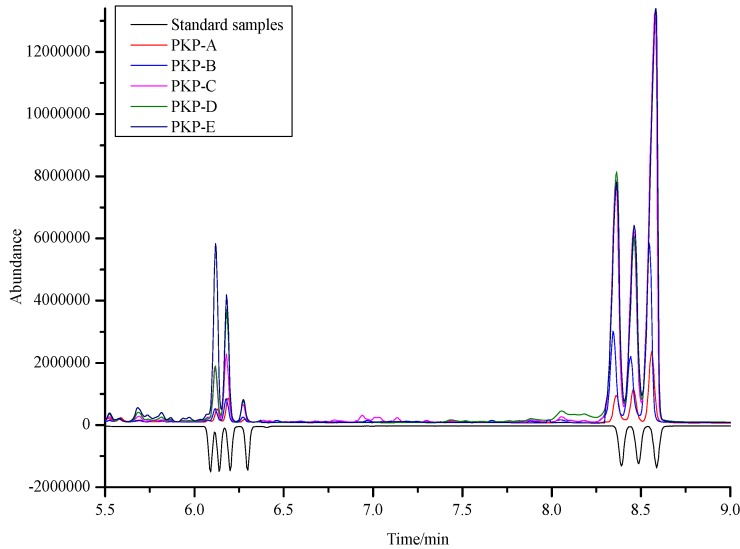
Monosaccharide composition of PKPs and standard samples (500 ng·mL^−^^1^).

Concerning monosaccharides’ quantitative and qualitative determination, the use of GC-MS is preferred because of its sensitivity. The GC-MS results (by Figure 1) indicated that the polysaccharides were composed of seven monosaccharides, including d-ribose, l-rhamnose, l-arabinose, d-xylose, d-mannose, d-glucose and d-galactose. The relative molar proportions of the seven monosaccharides were estimated and are shown in Table 3. These results showed that the PKP are composed of a large amount of d-mannose, d-glucose and d-galactose, and their contents are quite different from each other. Especially, the content of d-galactose was larger than that of other monosaccharides, followed by d-mannose, except in PKP-A.

**Table 3:** Molar proportions of seven monosaccharides of PKPs.

	Retention Time (%)	PKP-A	PKP-B	PKP-C	PKP-D	PKP-E
d-ribose	6.09	0.30	0.30	0.39	0.41	0.78
l-rhamnose	6.13	0.98	0.98	3.84	3.77	11.84
l-arabinose	6.20	1.96	1.85	5.01	8.70	9.84
d-xylose	6.29	0.40	0.53	0.12	1.84	1.96
d-mannose	8.39	2.16	7.77	21.98	24.47	23.60
d-glucose	8.48	2.75	4.80	16.02	15.41	16.59
d-galactose	8.58	5.11	12.75	36.93	36.69	36.95

### 2.2. FT-IR and UV Spectra

**Figure 2:**
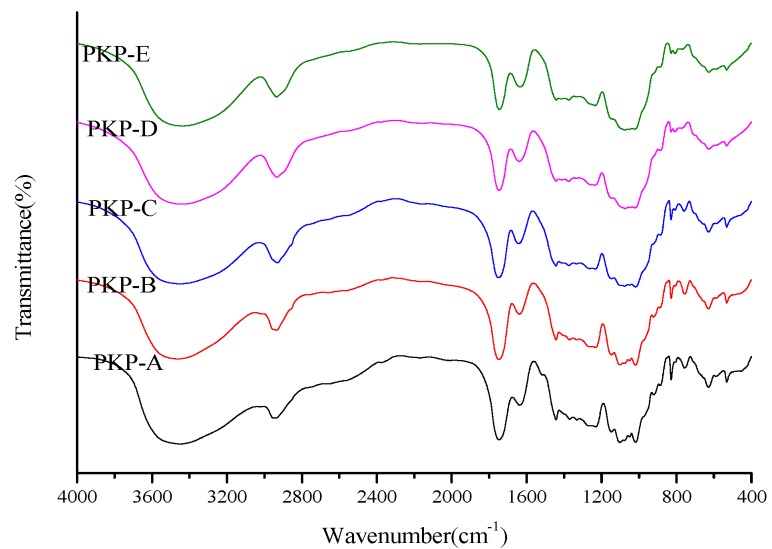
FT-IR spectra of the five PKPs.

FT-IR spectroscopy was used to study the carbohydrates due to its ability to identify main functional groups of plant sugars and complex carbohydrates. The FT-IR analysis obtained is presented in Figure 2. The stretching vibration of hydroxyl in the constituent sugar residues [16] can be easily recognized by the bands at 3446.0 cm^−1^. The spectra showed a prominent C-H stretching and bending vibration absorption [17] in the range of 2929–2939 cm^−1^. Thus the two bands are characteristic peaks of the polysaccharides. In addition, the high absorbance at 1,743 cm^−1^ is attributed to carbonyl group, and the small peak at 1637 cm^−1^ originated from associated water [18]. In the fingerprint region, the bands in the region of 1230 cm^−1^ are due to C-C stretching vibrations. Moreover, the bands towards 1100 cm^−1^ suggested that the peak was related to the stretching vibration of C-O. A small sharp and at 890 cm^−1^ was indicated as typical for β-anomers in PKPs [19]. The bands in 808.7 cm^−1^ indicated the presence of α-anomers [20]. From the above spectral assignments, the FT-IR spectra showed similar characteristics as the raw pine cones precipitated by ethanol solutions at different concentrations.

**Figure 3:**
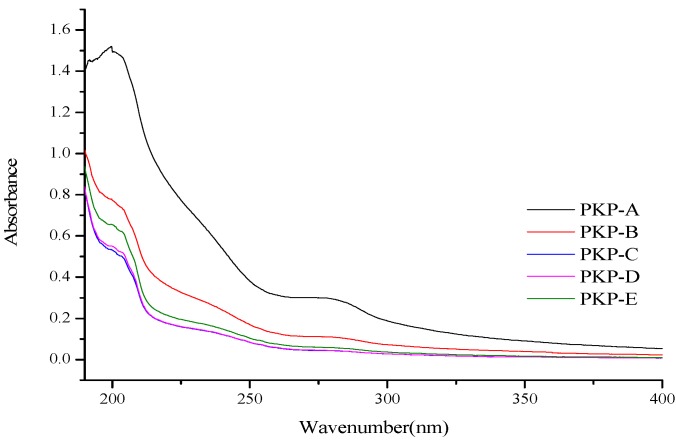
UV-vis spectra of PKPs.

The UV spectra of PKP fractions are shown in Figure 3. No absorption at 280 and 260 nm in the UV-vis spectrum were observed for any of the fractions, indicating a small amount of protein and the absence of nucleic acids [21]. The results were in accordance with the protein content results.

### 2.3. Antioxidant Activity

There are multiple recommended methods for measuring the antioxidant properties of materials to reflect their potential protective effects [12,22]. The hydroxyl radical detection and ABTS methods are reliable methods involved the determination of the disappearance of free radical with a spectrophotometer.

#### 2.3.1. Scavenging Effects on Hydroxyl Radical

Accumulating evidence strongly suggested that such stress is a vital causative factor of aging, brain dysfunction, liver diseases, cardiovascular disorder and carcinogenesis [23]. Among all ROS species, hydroxyl radical result in much of the oxidative damage to biomolecules [24]. Hydroxyl radical can easily lead to tissue damage or cell death. So the removal of hydroxyl radical is very important for the protection of living systems [25].

**Figure 4:**
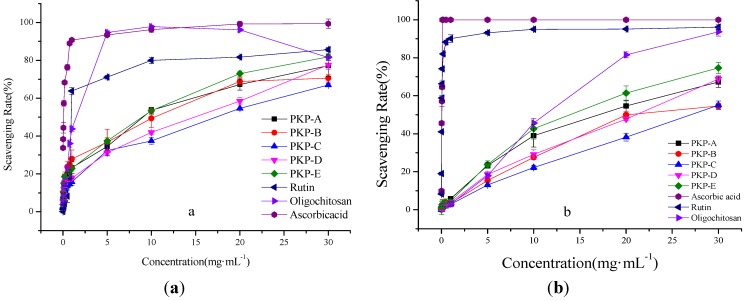
The *in vitro* antioxidant activity of the five fractions from pine cones. (**a**) Scavenging effects of ascorbic acid, rutin, oligochitosan and PKPs on hydroxyl radical; (**b**) Scavenging activities of ABTS radical by ascorbic acid, rutin, oligochitosan and PKPs.

Figure 4(a) showed the scavenging activities of the five fractions at different concentrations (0.01–30 mg·mL^−1^). It was clearly found that the scavenging rates increased with a rise in the sample concentration. The radical-scavenging ability of PKP-E was higher than that of others at higher concentrations, while they were almost at the same level at 1 mg·mL^−1^. At 30 mg·mL^−1^, the scavenging rate of PKP-A was nearly close to that of PKP-D, about 77%. Moreover, the positive control, ascorbic acid, showed the most effective activity on hydroxyl radical. The EC_50_ of ascorbic acid, rutin, oligochitosan and the five polysaccharides on hydroxyl radical are 0.058, 0.99, 0.11, 9.86, 9.69, 16.16, 14.43 and 9.02 mg·mL^−1^, respectively. These results proved that polysaccharides isolated from *P. koraiensis* had a definite effect on hydroxyl radical scavenging, and the PKP-E fraction showed the better effect.

#### 2.3.2. Scavenging Effects on ABTS Radical

The ABTS test is a simple, fast, reliable, inexpensive and also very adaptable tool to evaluate total antioxidant power of single compounds or complex mixtures [26]. After oxidation, ABTS·^+^ generates a blue-green radical cation. Samples with scavenging activity on ABTS radical are added into an ABTS solution, and the reaction will result in fading of the solution. Specific absorbance at 734 nm can be used in both organic and aqueous solvents as an index reflecting the antioxidant activity of extracted polysaccharides [27,28]. As Figure 4(b) shows, all of the tested samples showed dose-dependent activities. Moreover, PKP-E showed pronounced high radical scavenging ability. Furthermore, at 30 mg·mL^−1^, PKP-A and PKP-D showed the same level of antioxidant activity. The EC_50_ of ascorbic acid, rutin, oligochitosan and five fractions on ABTS radical are 0.079, 0.036, 11.03, 15.66, 21.58, 26.80, 20.06 and 13.38 mg·mL^−1^, respectively. These results indicated that PKP-E had strong ABTS radical scavenging power.

### 2.4. Inhibition Effects of the Polysaccharides on HepG2

Cancer is currently one of the leading causes of death. The lifetime probability of being diagnosed with cancer is more than 40% [29]. Natural dietary agents have drawn a lot of attention because of their potential to suppress cancers and to reduce risk of cancer development using decreasing oxidative stress. Accumulated evidence has demonstrated that polysaccharides have a broad spectrum of biological effects, such as antibiotic, antioxidant, anti-mutant, anticoagulant and immunostimulation activities [30,31,32]. The MTT assay was used to study the antiproliferative activity of PKP. MTT is reduced to an insoluble purple formazan by mitochondrial dehydrogenase. Cell proliferative activity was measured by comparison of the purple color formation. Dead cells, on the other hand, did not form the purple formazan due to their lack of the enzyme. 

**Figure 5:**
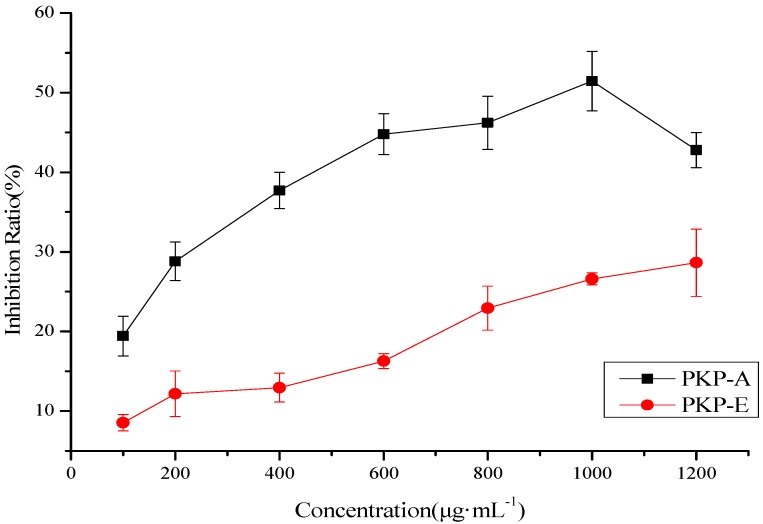
Inhibition rates of PKP-A and PKP-E on HepG2.

Cells were cultured for 72 h with several different levels of the polysaccharide fractions. At the concentration of 800 μg·mL^−1^, the inhibition rate of the five fractions were 46.20%, 22.21%, 20.71%, 21.30%, 22.93%, respectively. PKP-A and PKP-E showed better inhibitory activity than other samples. PKP-A and PKP-E were dissolved with culture solution to prepare a series of solutions (100, 200, 400, 600, 800, 1,000 and 1,200 μg·mL^−1^). Figure 5 shows the inhibition rates of PKP-A and PKP-E at increasing concentrations. The IC_50_ values of PKP-A and PKP-E were 1,072.5 and 2,070.0 μg·mL^−1^, respectively.

A close association exists between the activities of polysaccharides and the sugar composition, molecular weight, glycosidic linkage, comformation, degree of branching and so on [33]. It was reported that the bioactivities of polysaccharides were positively correlated with their molecular weight [34,35]. Many researchers have found that plant-derived polysaccharides composed of Rha, Gal and Ara had strong biological activities [36,37]. In the study, PKP-A and PKP-E displayed better biological activities. Among the five fractions, PKP-A had a much higher molecular weight than the other fractions, and PKP-E had the lowest one. In terms of the compositional analysis, the contents of Rha, Gal and Ara in PKP-E were much higher than others. Moreover, the contents of polyphenol in PKP were less than 1.2% (except for 3.5% in PKP-A, according to Table 1). The contents of polyphenol and the antioxidant activity did not show a dose-effect relationship; for instance, the polyphenol content of PKP-B was higher than PKP-E, while the scavenging activities of PKP-B were not better than those of PKP-E, which suggested that the existence of polyphenols had little effect on the antioxidant activity of PKP. According to the biological activities and preliminary characterization of the PKP results, we presume that the monosaccharide composition and molecular weight were crucial for the PKP bioactivities. 

## 3. Experimental

### 3.1. Materials and Chemicals

Pine cones of *Pinus koraiensis* used in the study were picked in a Korean pine forest in Yichun, Hei Longjiang, China. Trichloroacetic acid (TCA) was purchased from Tianjin Guangfu Fine Chemical Research Institute, Tianjin, China. Trifluoroacetic acid (TFA) was purchased from Dikma Technologies Inc., Beijing, China. 3-Phenylphenol was from Aldrich, Shanghai, China. d-Galacturonic acid, phenol, trypsin, penicillin-streptomycin solution, RPMI-1640 and dimethyl sulfoxide (DMSO), 3-(4,5-dimethylthiazol-2-yl)-2,5-diphenyltetrazolium bromide (MTT) were obtained commercially from Beijing Solarbio Science & Technology Co. Ltd., Beijing, China. Fetal bovine serum (FBS) was purchased from Zhejiang Sijiqing Biology Engineering and Material Co. Ltd., Hangzhou, China. The dextran standards were purchased from Sigma, Shanghai, China. Hepatoma cells (HepG2) was offered by the School of Food Science and Engineering of Harbin Institute of Technology, Harbin, China**.** All other reagents were of the highest available quality.

### 3.2. Extraction of Polysaccharides

The pine cones were refluxed to remove lipids with ethanol solution for 2 h twice. After filtering, the residues were extracted in boiling water (1:12, w/v) for 2 h [38]. The concentrated extract solution was precipitated with eight times volume of 95% ethanol overnight. The precipitate was collected by centrifugation at 3577 × g for 20 min, and then dried at reduced pressure, giving the crude polysaccharides.

### 3.3. Isolation and Purification of the Polysaccharides

#### 3.3.1. Removal of Protein from the Crude Polysaccharides

The crude polysaccharides contain proteins with large molecules, forming glycoproteins. Thus, removing protein is an important purification step. In order to get purer polysaccharides, we chose trichloroacetic acid (TCA) in this research [39]. The crude extract was dissolved in distilled water to make up a 0.5% polysaccharide solution. The above solution was mixed with 10% trichloroacetic acid (1:1, v/v). Then keep the mixture overnight at room temperature. The supernatant was harvested by decanting precipitate after centrifugation (3,577 × g, 20 min), dried by vacuum freezing dryer for follow-up tests.

#### 3.3.2. Graded Purification of the Polysaccharides

The polysaccharides solution was mixed with 95% ethanol, making the final concentration of ethanol 30% (v/v). The mixture was stirred and precipitated overnight at room temperature. The first fraction, collected by centrifugation at 3,577 × g for 10 min, was named PKP-A. The same method was used to deal with the supernatant. Certain volumes of 95% ethanol were add to the supernatant obtained by centrifugation and it was sequentially precipitated in ethanol solution with increased concentrations of 50%, 60%, 70%, 80% (v/v) of ethanol mixture [40], respectively. Thus, four other fractions named PKP-B, PKP-C, PKP-D and PKP-E were collected. The five fractions were dried by vacuum freezing for subsequent treatments.

### 3.4. Determination of Molecular Weight

The molecular weight of the five fractions were determined by gel-permeation chromatography (GPC, Agilent Technologies, Santa Clara, CA, USA) [41], in combination with a high-performance liquid chromatography (HPLC) instrument (Agilent 1100) equipped with an Agilent G1362A Refractive Index Detector. Standard dextrans (M_W_ 125 × 10^4^, 96.5 × 10^4^, 12.5 × 10^4^, 2.58 × 10^4^ and 0.41 × 10^4^, respectively) were passed through the column. Five purified fractions (10.0 mg) were dissolved in ultrapure water (10 mL), separately passed through 0.45 μm filters, and applied to the gel column. The samples were eluted with ultrapure water at a flow rate of 1.0 mL·min^−1^, maintained at a temperature of 25 °C. The molecular weights were calculated by reference to a standard curve plotted according to the retention time and the logarithm of the standard dextrans’ respective molecular weights [42].

### 3.5. Monosaccharide Composition and Properties

#### 3.5.1. Hydrolysis of the Five Polysaccharides

The five fractions (15 mg) were dissolved in 2 M TFA (4 mL) and hydrolyzed at 110 °C for 4 h in sealed glass tubes. Then the hydrolyzed products were evaporated to dryness at reduced pressure at 50 °C. The residue were dissolved in 3 mL methanol and dried by a stream of nitrogen gas. In order to remove TFA completely, the treatment was repeated 3–4 times.

#### 3.5.2. Derivatization of the Monosaccharides

Because of the nonvolatile nature of carbohydrates, they must be transformed to volatile derivatives for GC determination. The hydrolyzed products were dissolved in 3 mL distilled water, and mixed with sodium borohydride (25 mg). The reduction reaction was performed at room temperature for 3 h by intermittent oscillation. A few drops of glacial acetic acid were added to end the reaction until no bubbles appeared. The resulting solution was evaporated to dryness under reduced pressure at 50 °C. Methanol (3 mL) and a stream of nitrogen gas were used to remove the reducing agent 3–4 times, then the residue was dried at 110 °C for 15 min [43]. The derivatives were mixed with pyridine (1 mL) and acetic anhydride (3 mL). The reaction was carried out in water bath at 100 °C for 3–5 h. The mixture was evaporated to dryness at 80 °C and trichloromethane (5 mL) was added to dissolve the residue. Isopyknic distilled water was used to wash the organic solution three times to remove ions or extra acetic anhydride. Finally, the water was removed by adding anhydrous sodium sulfate. The supernatant was filtered through a 0.45 μm membrane prior to GC-MS analysis.

#### 3.5.3. GC-MS Analysis

A Gas Chromatograph-Mass Spectrometer (GC-MS, Agilent HP 6890-5973, USA) was used for identification and quantification, equipped with capillary column DB-5(60 m × 0.25 mm I.D., 0.25 μm film thickness). Helium was carrier gas at a flow rate of 1.0 mL·min^−1^. The temperature was programmed as follows: initial temperature 150 °C, maintained 1 min; rise to 250 °C at a rate of 25 °C·min^−1^, retained for 10 min. The total analysis was conducted in 12 min and the equilibration time was 2 min. The temperature of the injection port was 250 °C and 1 μL sample was injected in splitless mode. The mass spectrometer was operated in electron ionization mode at an ionizing energy of 70 eV, the temperature of ion source 230 °C. The detector was used to scan from *m/z* 50 to 500.

### 3.6. Neutral Sugar, Uronic Acid, Polyphenol, Protein and Amino Acid Analysis

The contents of neutral sugar were determined by the phenol-sulfuric acid test [44] with slight modifications, taking d-glucose as the standard at 490 nm. Uronic acid contents were determined by photometry with *m*-hydroxybiphenyl at 525 nm with d-galacturonic acid as the standard [45]. The phenolic contents were measured as described by Rajkumar *et al.* [46] with a little modification. Coomassie brilliant blue method [47] was used to measure the protein levels of the five fractions. Amino acids in the five fractions were determined after acid hydrolysis with 6 M HCl at 110 °C for 24 h in sealed glass tubes, as described by Haimei Li and others [48]. After neutralization and dissolving to constant volume, the sample was passed through 0.45-μm filter and analyzed by a Hitachi l-8800 amino acid analyzer.

### 3.7. FT-IR and UV Spectroscopy

FT-IR spectra of the samples were recorded on a Fourier-transform infrared spectrophotometer (PerkinElmer, Norwalk, CT, USA). Dried polysaccharides were ground and pelletized with KBr. To compare structures of the graded fractions, their spectra were recorded within a range of 400–4,000 cm^−1^ [49]. The ultraviolet spectra of the fraction solutions (1.0 mg·mL^−1^) were recorded with a UV-vis spectrophotometer (TU-1900 double beam UV-visible light spectrophotometer, Beijing, China) in the the 190-400 nm region in 1.00 cm quartz cell against distilled water as the blank.

### 3.8. Antioxidant Activity of the Polysaccharides

#### 3.8.1. Hydroxyl radical scavenging assay

The hydroxyl radical assay was measured by Jen’s method [50] with a slight modification. Samples were dissolved in distilled water (0.01–30 mg·mL^−1^). The sample solution (1.0 mL) was mixed with FeSO_4_ (9 mM, 1.0 mL) and 9 mM salicylic acid solution (1.0 mL, 50% ethanol). Then 8.8 mM H_2_O_2_ (1.0 mL) was added to start the reaction. The mixture was kept in water bath at 37 °C for 1 h. The background was mixed as described above except 50% ethanol (1.0 mL) was used in place of the salicylic acid solution. For the control distilled water (1.0 mL) was substituted for the polysaccharide solution. After warming in a water bath, the absorbance of the mixture was measured at 510 nm. The hydroxyl radical scavenging rate was calculated using the following formula:

Scavenging Rate (%) = [1 − (A_1_ − A_2_)/A_0_] × 100% (1)

where A_0_ is the absorbance of the control group (without polysaccharides), A_1_ is the absorbance of the test group, A_2_ is the absorbance of the background group.

#### 3.8.2. ABTS Radical Scavenging Assay

According to the method describe by Hai-Chao Zhou *et al*. [51], the radical scavenging activity against the ABTS·^+^ radical was determined. ABTS (50 mL, 7 mM) was mixed with 140 mM potassium peroxydisulfate (890 μL), then kept in dark at room temperature for 12–16 h before use. The samples were prepared in a variety of concentrations (0.01–30 mg·mL^−1^). The polysaccharides solution (0.2 mL) was added to the ABTS·^+^ solution. The absorbance of the mixture was measured at 734 nm namely after holding at room temperature for 6 min. The scavenging activity was calculated by the following equation:

Scavenging Rate (%) = [1 − (A_1_ − A_2_)/A_0_] × 100% (2)

where A_0_ is the absorbance of the control group, A_1_ is the absorbance of the test group, A_2_ is the absorbance of the background group (without ABTS·^+^).

### 3.9. Anti-Hepatoma Activity Assay

The anticancer activity of polysaccharides was first recognized by Nauts *et al.* when they found the certain polysaccharides could induce complete remission in patients with cancer in 1949 [52].

#### 3.9.1. Cell Culture

The HepG2 (a human hepatoma cell line) was used to determine the activity of samples. The medium used for hepatoma cells was prepared with RMPI-1640 supplemented with streptomycin (1,000 U·mL^−1^), penicillin (1,000 μg·mL^−1^) and 10% fetal bovine serum [53]. The culture bottles were placed in a humidified in 5% CO_2_ atmosphere at a temperature of 37 °C. The medium was changed every 24 h. At 90% confluence, the cells were trypsinized with 0.25% trypsin in PBS solution for 1 min and resuspended in culture medium.

#### 3.9.2. Preliminary MTT Analysis

After incubation of HepG2 cells, the MTT assay [54] was used to measure cell viability. After the digestion by 0.25% trypsin digestion fluid, cell suspension was prepared with culture solution. The cell broth was transferred to 96-well plates, and 100 μL of cell broth was injected into the well. After culturing at 37 °C, 5% CO_2_ for 24 h, the control and test groups (200, 400, 800 μg·mL^−1^, final concentrations) were added to the wells. The cells were incubated under the same conditions. After 72 h, MTT (5 mg·mL^−1^, 10 μL) was mixed with the medium. Then the 96-well plates were kept at 37 °C for 4 h. After that, the medium was discarded and DMSO (100 μL) was inject into each well. The absorbance was measured at 490 nm by an automatic microplate reader. The inhibition ratio was calculated as the following equation:

Inhibition Ratio (%) = (1 − A_1_/A_0_) × 100% (3)

where A_0_ is the absorbance of test group, A_1_ is the absorbance of control group.

#### 3.9.3. Further MTT Assay

After the screening test above, PKP-A and PKP-E were proved to inhibit HepG2 cells more effectively than the other fractions. Therefore, further assay about the samples of inhibition activity was needed. The polysaccharides were dissolved with culture solution to prepare a series of solutions (100, 200, 400, 600, 800, 1,000 and 1,200 μg·mL^−1^). The most effective concentration was determined by the MTT assay.

## 4. Conclusions

The objective of our study was to evaluate and compare the chemical characteristics and bioactive properties of graded polysaccharides from pine. In the study, five fractions were successfully isolated from the crude polysaccharides of pine cones of *Pinus koraiensis* by increasing the concentration of ethanol during precipitation. According to the results above, it was concluded the contents of neutral sugar, protein and seven monosaccharides were different from each other. In addition, the molecular weights of the five polysaccharides were increasingly decreasing. Antioxidative tests proved that PKP-E was the most effective on hydroxyl radical or ABTS radical, reaching around 80% scavenging. Finally, PKP-A and PKP-E were found to behave better activity in inhibition of HepG2. The IC_50_ values of PKP-A and PKP-E were 1,072.5 and 2,070.0 μg·mL^−1^, respectively. Our results can give guidance for identifying sources of antioxidant compounds. Meanwhile, the correlation between chemical characteristics and antioxidant properties for the purified polysaccharide fractions is ongoing in our lab.
